# Development and Characterization of a Novel FVB-*Prkdc^R2140C^* Mouse Model for Adriamycin-Induced Nephropathy

**DOI:** 10.3390/genes15040456

**Published:** 2024-04-04

**Authors:** Masaki Watanabe, Yuki Ishii, Kazuki Hashimoto, Hayato R. Takimoto, Nobuya Sasaki

**Affiliations:** Laboratory of Laboratory Animal Science and Medicine, School of Veterinary Medicine, Kitasato University, Towada 034-8628, Japan

**Keywords:** Adriamycin, chronic kidney disease, FVB/N, genetic background, mouse, nephropathy, *Prkdc*

## Abstract

The Adriamycin (ADR) nephropathy model, which induces podocyte injury, is limited to certain mouse strains due to genetic susceptibilities, such as the *Prkdc^R2140C^* polymorphism. The FVB/N strain without the R2140C mutation resists ADR nephropathy. Meanwhile, a detailed analysis of the progression of ADR nephropathy in the FVB/N strain has yet to be conducted. Our research aimed to create a novel mouse model, the FVB-*Prkdc^R2140C^*, by introducing *Prkdc^R2140C^* into the FVB/NJcl (FVB) strain. Our study showed that FVB-*Prkdc^R2140C^* mice developed severe renal damage when exposed to ADR, as evidenced by significant albuminuria and tubular injury, exceeding the levels observed in C57BL/6J (B6)-*Prkdc^R2140C^*. This indicates that the FVB/N genetic background, in combination with the R2140C mutation, strongly predisposes mice to ADR nephropathy, highlighting the influence of genetic background on disease susceptibility. Using RNA sequencing and subsequent analysis, we identified several genes whose expression is altered in response to ADR nephropathy. In particular, *Mmp7*, *Mmp10*, and *Mmp12* were highlighted for their differential expression between strains and their potential role in influencing the severity of kidney damage. Further genetic analysis should lead to identifying ADR nephropathy modifier gene(s), aiding in early diagnosis and providing novel approaches to kidney disease treatment and prevention.

## 1. Introduction

Chronic kidney disease (CKD) results from a variety of primary diseases, including diabetic nephropathy, chronic glomerulonephritis, and renal sclerosis, and leads to long-term, irreversible changes in kidney function and structure [1]. More than 2.5 million people are currently receiving renal replacement therapy, and this is expected to increase to 5.4 million by 2030 [2,3]. In addition, CKD is closely associated with major diseases such as diabetes, hypertension, and heart disease, making it a major health and cost burden for which there is no curative treatment [4,5,6]. The development of curative treatments is critically dependent on the availability of animal models that can mimic human CKD. Presently, many CKD models have been generated, which play an essential role in understanding the mechanisms behind multifactorial CKD in developing new treatments and improving existing therapies.

The Adriamycin (ADR) nephropathy model, which can induce podocyte injury with a single dose of ADR, mimics human primary glomerular diseases such as focal segmental glomerulosclerosis (FSGS), which is characterized by severe proteinuria [7]. FSGS is a common pathological form of refractory nephrotic syndrome with diverse causes, including circulating factors, genetic mutations, infections, toxicities, or adaptive responses [8,9,10]. The simplicity and reproducibility of the ADR model have made it one of the most widely used tools for investigating the processes underlying the development of human FSGS and CKD [11]. However, using the ADR nephropathy model has been limited to certain mouse strains, including the BALB/c strain [12,13]. Genetic analysis of the BALB/c strain suggested that susceptibility to ADR is due to a C6418T mutation in exon 49 of the *Prkdc* (DNA-dependent protein kinase catalytic subunit) gene, resulting in an R2140C polymorphism [14]. Therefore, we generated C57BL/6J (B6)-*Prkdc^R2140C^* mice with the R2140C mutation introduced by CRISPR/Cas9, assessed their susceptibility to ADR, and experimentally demonstrated that the PRKDC mutation is the causative mutation for ADR nephropathy [15]. It has now been shown that modifier genes influence the severity of ADR nephropathy, with strain differences observed, and the causative gene has been proposed to be located between *D9Mit182* and *D9Mit229* on chromosome 9 in B6, accounting for approximately 30% of the reduction in the histological damage score. However, the causative gene has yet to be identified [14].

The FVB/N strain, widely used in renal disease research, is highly susceptible to nephropathy in several renal disease models, including HIV-associated nephropathy, 75% nephrectomy, and CD151 knockout mice [16,17,18]. However, the FVB/N strain without the R2140C mutation resists ADR nephropathy. Meanwhile, a detailed analysis of the progression and severity of ADR nephropathy has yet to be performed. Thus, in this study, we generated congenic mice by introducing the *Prkdc^R2140C^* allele of B6-*Prkdc^R2140C^* into the FVB/N genetic background and investigated whether there was a difference in the severity of ADR nephropathy compared with the BALB/c and B6 strains.

## 2. Materials and Methods

### 2.1. Ethical Statement

The research was conducted in accordance with Kitasato University’s regulations for the care and use of laboratory animals. The President of Kitasato University approved the animal experiment protocol through the judgment of the Institutional Animal Care and Use Committee of Kitasato University (Approval ID: 22-059). A humane endpoint was used when mice exhibited moribundity or severe weight loss (20% body weight loss within a few days).

### 2.2. Animals

As previously described, B6-*Prkdc^R2140C^* mice were generated in the C57BL/6J strain [15]. BALB/c (BALB/cByJJcl), and FVB/N (FVB/NJcl) mice were obtained from CLEA Japan (Tokyo, Japan). The animal facilities were air-conditioned, and the mice were maintained at 22 ± 2 °C with 40–60% relative humidity under a 12 h light/dark cycle. Standard laboratory chow CE-2 (CLEA Japan) and tap water were provided ad libitum.

### 2.3. Generation of Congenic FVB-Prkdc^R2140C^ Mice

B6-*Prkdc^R2140C^* and FVB/N mice were crossed to produce F1 mice carrying the heterozygous *Prkdc^R2140C^* mutation. These mice were backcrossed to FVB/N mice, and this process of backcrossing and genotyping was repeated for 10 generations to establish a congenic strain. Finally, by mating heterozygous mice and through genotyping, *Prkdc^R2140C^* homozygous mice (FVB-*Prkdc^R2140C^*) were produced. The mutant genotype was determined by PCR according to our previous publication [15].

### 2.4. Administration of ADR

ADR was administered to different strains of 8-week-old male mice (B6-*Prkdc^R2140^*, BALB/c, FVB-*Prkdc^R2140C^*). ADR (doxorubicin hydrochloride, Sigma-Aldrich, St. Louis, MO, USA) was dissolved in saline (Otsuka Pharmaceutical, Tokyo, Japan) to prepare a 2 mg/mL solution, which was then administered to the mice. To assess the susceptibility of each strain to ADR, a 13 mg/kg dose was administered intravenously. Urine and kidneys were collected one week after dosing to assess acute kidney injury. In addition, urine and kidneys were collected 28 days after administration of ADR at 13 mg/kg to assess chronic kidney injury. The control group received an equal volume of normal saline. Mice were euthanized by exsanguination through the posterior vena cava after administration of a mixed anesthetic consisting of medetomidine hydrochloride (0.75 mg/kg, Nippon Zenyaku Kogyo, Fukushima, Japan), midazolam (4 mg/kg, Sandoz Ltd., Tokyo, Japan), butorphanol tartrate (5 mg/kg, Meiji Seika Pharma Co., Ltd., Tokyo, Japan), and saline (Otsuka Pharmaceutical, Tokyo, Japan) in a volume of 10 mL per kg body weight, subcutaneously injected [19]. The kidneys were then excised post-mortem.

### 2.5. Detection of Urinary Albumin

Urinary albumin (Alb) was collected from the bladder 1 and 4 weeks after ADR administration and detected by SDS-PAGE. As a positive control, 5 μg of bovine serum albumin was loaded simultaneously. After electrophoresis, the gel was stained with Coomassie Brilliant Blue (CBB) using a Rapid Stain CBB kit (Nacalai Tesque, Kyoto, Japan) and destained in distilled water (DW) for 24 h. The staining image was captured using a commercial scanner (GTX-820, EPSON, Nagano, Japan).

### 2.6. Calculation of the Urinary Albumin-to-Creatinine Ratio

The amount of albumin in the collected urine was measured by ELISA (LBIS Mouse Albumin ELISA kit, Fujifilm Wako Pure Chemical Corp., Osaka, Japan). In addition, urinary creatinine (Cre) levels were measured in the same urine samples using a Creatinine Colorimetric Assay kit (Cayman Chemical, MI, USA), according to the manufacturer’s instructions. The measured albumin levels were corrected for the urinary Cre levels, and the albumin/Cre ratio (Alb/Cre) was calculated.

### 2.7. Histology

Harvested kidneys were fixed in 4% paraformaldehyde and embedded in paraffin. Sections were prepared in thicknesses of 2 µm and 4 µm from the paraffin blocks for periodic acid-Schiff (PAS) and picrosirius red staining, respectively. The kidneys were used for PAS staining 7 days after ADR administration. According to established protocols [20], sections were treated with 0.5% periodic acid solution for 5 min, washed in distilled water, stained with Schiff’s reagent (Fujifilm Wako Pure Chemical Corporation, Osaka, Japan) for 30 min, washed thrice with 0.8% sodium bisulfite solution for 3 min each, rinsed in running water, counterstained with hematoxylin for 4 min, washed in running water, rinsed in distilled water, cleared, and mounted. Kidneys were used for picrosirius red staining 28 days after ADR administration. According to previous studies [20], sections were deparaffinized, washed in running water, rinsed in distilled water for 1 min, stained with solution A (0.5 g Sirius Red in 500 mL picric acid saturated solution) for 1 h, treated thrice with solution B (5 mL acetic acid in 1000 mL distilled water) for 3 s each, cleared, and mounted. Fibrosis was quantified using picrosirius red stained tissue sections. The heights of the tubules in the renal cortex were measured in three randomly selected fields (×100 magnification) using Image J software version 1.52a (National Institutes of Health, Bethesda, MD, USA). The average value per 50 tubules was calculated. Fibrotic area quantification was performed by taking three randomly selected fields (×40 magnification) in the renal cortex, and the stained areas were measured using Image J software version 1.52a (National Institutes of Health, Bethesda, MD, USA) to calculate the percentage of renal section area. Glomeruli and vessels stained with picrosirius red were excluded from the measurement assessing tubulointerstitial fibrosis.

### 2.8. RNA Sequencing Analysis

Kidneys from each experimental group were used for RNA extraction according to the procedure provided with the NucleoSpin^®^ RNA kit (Takara Bio, Shiga, Japan). The extracted RNA was used for RNA sequencing (RNA-seq) analysis. This analysis was performed by the contract analysis service of GENEWIZ, Inc. (Tokyo, Japan) using the Illumina NovaSeq system (2 × 150 bp configuration, 6 Gb per sample). Specifically, sequencing was performed on the NovaSeq 6000 system (Illumina, San Diego, CA, USA) using a poly-A selection method for strand-specific RNA-seq analysis. Gene expression analysis was performed using the Bioconductor DESeq2 package, a model based on the negative binomial distribution, to perform differential expression gene analysis. Genes were considered differentially expressed if they had an adjusted *p*-value (Padj) of <0.01.

### 2.9. Quantitative Reverse Transcription-PCR (qRT-PCR)

cDNA was synthesized from RNA using ReverTra Ace (Toyobo, Osaka, Japan). qRT-PCR was performed using Thunderbird SYBR qPCR Mix (Toyobo, Osaka, Japan), according to the manufacturer’s instructions. The reaction was analyzed using CFX Maestro (Bio-Rad Laboratories Inc., Hercules, CA, USA). The PCR primer sequences used in this study were acquired from previous studies [21,22,23,24,25,26,27] and are listed in Table 1.

### 2.10. Statistics

All data are presented as mean ± standard deviation (SD). A one-way analysis of variance (ANOVA) was used to statistically analyze parametric data among multiple groups, followed by the Tukey–Kramer post hoc test for pairwise comparisons. A *p*-value < 0.05 was considered statistically significant. All statistical analyses were performed using JMP Pro 17 (SAS Institute, Cary, NC, USA).

## 3. Results

### 3.1. Urinary Alb Excretion

**Figure 1:**
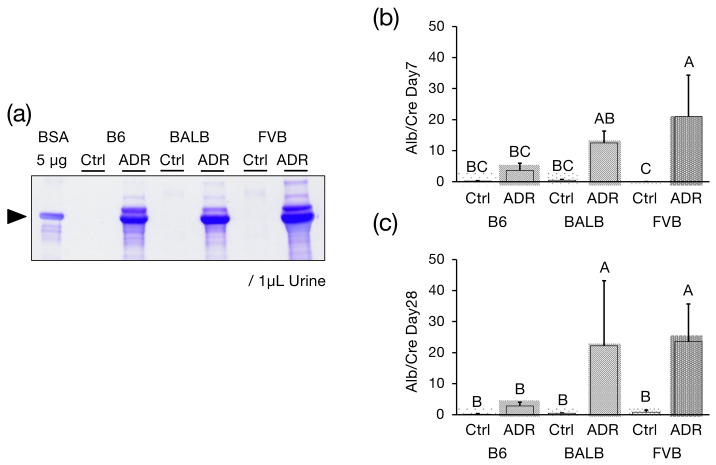
Urinary albumin excretion in Adriamycin (ADR)-treated B6-*Prkdc^R2140C^* (B6), BALB/c (BALB), and FVB-*Prkdc^R2140C^* (FVB) mice. (**a**) SDS-PAGE analysis of representative individual B6-*Prkdc^R2140C^*, BALB/c, and FVB-*Prkdc^R2140C^* mice. Bovine serum albumin: BSA (**b**) Urinary albumin excretion in control B6-*Prkdc^R2140C^* (n = 5), BALB/c (n = 4), and FVB- *Prkdc^R2140C^* (n = 7), as well as in B6-*Prkdc^R2140C^* mice (n = 8), BALB/c mice (n = 5), and FVB-*Prkdc^R2140C^* mice (n = 8), 7 days after ADR administration. (**c**) Urinary albumin excretion in control B6-*Prkdc^R2140C^* (n = 6), BALB/c (n = 4), and FVB-*Prkdc^R2140C^* (n = 5) mice and in ADR-treated B6-*Prkdc^R2140C^* (n = 8), BALB/c (n = 5), and FVB-*Prkdc^R2140C^* (n = 5) mice, 28 days after ADR administration. A vs. B: *p* < 0.05. A vs. C: *p* < 0.05. A vs. BC: *p* < 0.05.

To evaluate the development of glomerular injury induced by ADR administration, ADR was administered to a congenic strain, FVB-*Prkdc^R2140C^*, created by backcrossing the F1 mice to FVB/N mice for 10 generations and identifying genotypes. In this study, the weight of the 8-week-old male FVB-*Prkdc^R2140C^* mice was reported as 25.35 ± 1.050 (n = 8), while the FVB-*Prkdc^WT^* mice weighed 25.45 ± 1.030 (n = 8), meaning no significant difference was noted in observed weights between the two groups. Additionally, under normal breeding conditions, there was minimal albumin leakage into the urine of the FVB-*Prkdc^R2140C^* mice. In ADR nephropathy mice, proteinuria peaks on day 7 [28]; therefore, albumin in urine collected on day 7 after SDS-PAGE confirmed treatment, and albumin was detected in all three strains: B6-*Prkdc^R2140C^*, BALB/c, and FVB-*Prkdc^R2140C^* (Figure 1a). Subsequently, the amount of albumin in the urine collected on days 7 and 28 was measured by ELISA and corrected for Cre levels. On day 7, FVB-*Prkdc^R2140C^* had a significantly higher Alb/Cre ratio than B6-Prkdc^R2140C^ (Figure 1b), and on day 28, both BALB and FVB-*Prkdc^R2140C^* had a significantly higher Alb/Cre ratio than B6-*Prkdc^R2140C^* (Figure 1c).

### 3.2. Histological Investigation

**Figure 2:**
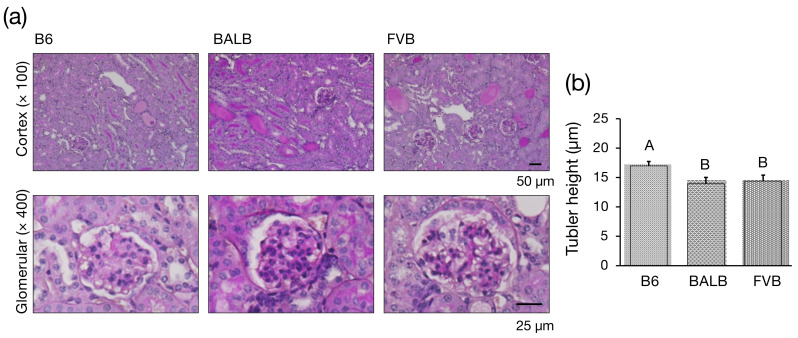
Histological analysis of kidneys from Adriamycin (ADR)-treated mice. (**a**) Representative periodic acid-Schiff-stained renal sections from ADR-treated mice. B6-*Prkdc^R2140C^*: B6, BALB/c: BALB, FVB-*Prkdc^R2140C^*: FVB. (**b**) Tubular height. ADR-treated B6-*Prkdc^R2140C^* (n = 4), BALB/c (n = 4), and FVB-*Prkdc^R2140C^* (n = 4) mice were analyzed. A vs. B: *p* < 0.05.

In ADR nephropathy mice, proteinuria peaks on day 7 after administration, followed by a gradual decrease in the number of podocytes per glomerulus and increased sclerotic glomeruli [28]. Therefore, pathological histological images obtained by PAS staining one week after ADR administration were compared between the different strains. In B6-*Prkdc^R2140C^*, BALB/c, and FVB-*Prkdc^R2140C^*, mild dilatation of mesangial areas indicating glomerular damage and tubular damage (dilatation of tubular lumen, atrophy, flattening, and formation of tubular casts) were observed (Figure 2a). Tubular atrophy is a hallmark feature of CKD that indicates the extent of tubular damage, which can be quantitatively assessed by measuring the height of the proximal tubules. This measurement serves as a valuable marker to evaluate the severity of the tubular injury [29]. After administering ADR to induce nephropathy, an experiment was conducted to measure and compare the heights of the tubules in the different mice strains to assess the extent of the tubular damage. In BALB/c and FVB-*Prkdc^R2140C^* mice, there was a significant reduction in the height of the tubules (Figure 2b), indicating a marked degree of tubular injury in these strains following ADR administration.

### 3.3. Assessment of Interstitial Fibrosis

**Figure 3:**
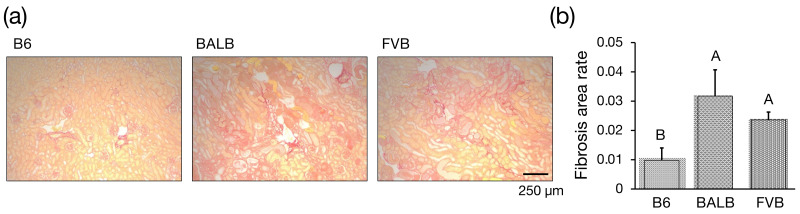
Histological Figure 3. Interstitial fibrosis with picrosirius red staining. (**a**) Representative picrosirius-red-stained images of kidney sections from Adriamycin (ADR)-treated mice. B6-*Prkdc^R2140C^*: B6, BALB/c: BALB, FVB-*Prkdc^R2140C^*: FVB. (**b**) Fibrosis area vs. total area. ADR-treated B6-*Prkdc^R2140C^* (n = 4), BALB/c (n = 4), and FVB-*Prkdc^R2140C^* (n = 4) mice were analyzed. A vs. B: *p* < 0.05.

Tubulointerstitial fibrosis is the most common pathological finding in CKD and contributes significantly to decreased renal function [30]. Measurement of the fibrotic area using picrosirius red staining showed that B6-*Prkdc^R2140C^*, BALB/c, and FVB-*Prkdc^R2140C^* mice all exhibited tubulointerstitial fibrosis 28 days after ADR administration. Notably, the areas of fibrosis in BALB/c and FVB-*Prkdc^R2140C^* mice were significantly larger than those in B6-*Prkdc^R2140C^* mice (Figure 3a,b).

### 3.4. Analysis of Differentially Expressed Genes through RNA-seq

**Figure 4:**
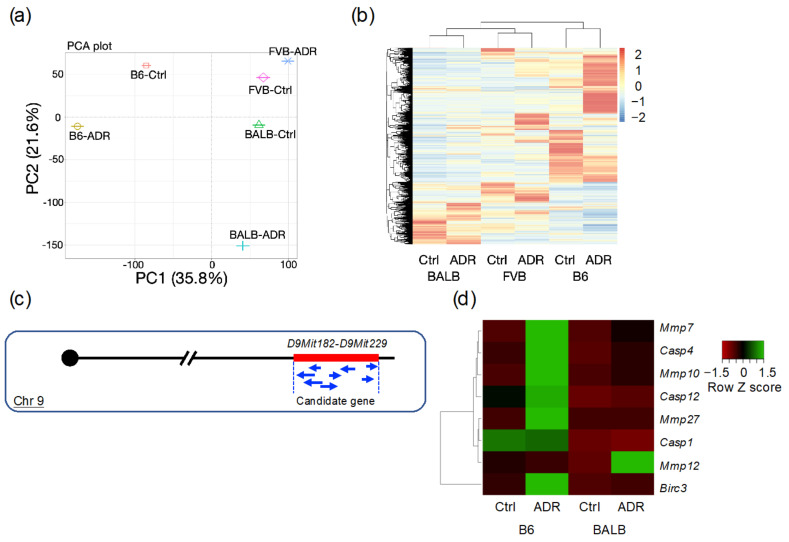
RNA sequencing analysis. (**a**) PCA analysis. (**b**) Heatmap of clustering analysis. (**c**) Overview of kidney disease resistance genes between C57BL/6 and BALB. (**d**) Heatmap of differentially expressed genes in the resistance gene locus. Analyses were performed with n = 1 for each strain. B6-*Prkdc^R2140C^*: B6, BALB/c: BALB, FVB-*Prkdc^R2140C^*: FVB.

One week after ADR administration to induce nephropathy, significant differences in the tubular damage severities were observed between the different mouse strains. Therefore, kidneys from this time point were used for RNA-seq analysis. First, dimension reduction and comparison of the RNA-seq data by principal component analysis (PCA) revealed significant differences in gene expression profiles between the strains (Figure 4a). Subsequently, genes that showed expression changes were clustered and visualized in a heatmap, as shown in Figure 4b and Supplementary Table S1. These results suggest that significant differences in gene expression profiles between strains characterize ADR-induced nephropathy. Conversely, the severity of ADR-induced nephropathy is influenced by modifier genes, and the difference in susceptibility between B6 and BALB/c strains has been reported to be located between D9Mit182 and D9Mit229 on chromosome 9 (Figure 4c) [14]. Eight genes showing significant expression changes were identified within this locus that showed expression variations: *Mmp7*, *Casp4*, *Mmp10*, *Casp12*, *Mmp27*, *Casp1*, *Mmp12*, and *Birc3* (Figure 4d).

### 3.5. Analysis of Differentially Expressed Genes by qRT-PCR

**Figure 5:**
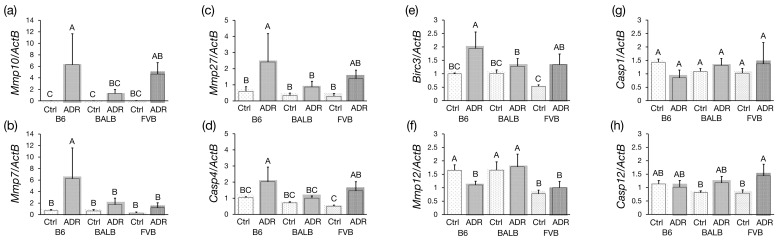
Expression analysis of differentially expressed genes in the resistance locus after administration of Adriamycin (ADR). Analyses were performed with n = 5 for each strain. B6-*Prkdc^R2140C^*: B6, BALB/c: BALB, FVB-*Prkdc^R2140C^*: FVB. A vs. B: *p* < 0.05. A vs. C: *p* < 0.05. B vs. C: *p* < 0.05. A vs. BC: *p* < 0.05.

qRT-PCR validated the expression levels of these eight identified genes. The expressions of *Mmp10*, *Mmp7*, *Mmp27*, *Casp4*, and *Birc3* were significantly higher in the ADR-treated B6-*Prkdc^R2140C^* mice compared to ADR-treated BALB/c mice, while *Mmp12* expression was significantly decreased (Figure 5a–f). Conversely, the expression levels of *Casp1* and *Casp12* in B6-*Prkdc^R2140C^* were comparable to those in BALB/c mice (Figure 5g,h). In addition, *Mmp7* expression was significantly higher in ADR-treated B6-*Prkdc^R2140C^* mice than in ADR-treated FVB-*Prkdc^R2140C^* mice.

## 4. Discussion

Our previous research aimed to adapt the ADR nephropathy model for use in the standard B6 mouse strain. To achieve this, we used CRISPR/Cas9 technology to introduce the R2140C mutation, a polymorphism between the B6 and BALB strains, to create the B6-*Prkdc^R2140C^* mouse. Our findings demonstrate that the susceptibility to Adriamycin nephropathy in BALB/c mice is attributed to the R2140C mutation in the PRKDC gene [15]. Conversely, the FVB/N strain, which does not possess the R2140C mutation and is widely used in renal disease research, is resistant to ADR nephropathy [13]. However, detailed analyses of the progression and severity of nephropathy in this strain have yet to be performed. Therefore, we generated a congenic mouse model in the current study by introducing the *Prkdc^R2140C^* allele from B6-*Prkdc^R2140C^* into the FVB/N strain genetic background. This was performed to investigate whether there are differences in the severity of ADR nephropathy compared to the BALB/c and B6 strains. This approach aims to elucidate the effect of genetic background on the onset and severity of ADR nephropathy.

We measured urinary Alb levels and performed histopathological analyses on day 7 following ADR administration to evaluate the sensitivity of the strain to ADR nephropathy. Our findings revealed that FVB-*Prkdc^R2140C^* mice exhibited pronounced albuminuria by day 7 post-ADR treatment, indicating a severe response. Compared with the established ADR-sensitive strains, such as B6-*Prkdc^R2140C^*, the FVB-*Prkdc^R2140C^* mice showed a significant increase in the Alb/Cre ratio, suggesting an increased susceptibility to glomerular damage. The histopathological examination further supported these findings, where the tubular height metric—a marker of tubular damage—was significantly reduced in FVB-*Prkdc^R2140C^* mice compared to the B6-*Prkdc^R2140C^* mice, indicating pronounced tubular damage. This observation suggests that the FVB/N strain has an increased susceptibility to glomerular damage and a high sensitivity to tubular damage. The high sensitivity of the FVB/N strain has further been confirmed in various models of renal disease. For instance, *Tns2* knockout mice, which develop nephrotic syndrome in a strain-dependent manner, present with proteinuria due to glomerular basement membrane abnormalities and subsequent podocyte foot process effacement as early as two weeks of age in the FVB/N background. Conversely, the B6 strain completely resists this phenotype [31,32]. Investigations into *Tns2* knockout mice have suggested that the FVB/N strain is susceptible to both glomerular and tubular damage, with potential causative genes located on chromosomes 2 and 10 [33], although their exact identification remains elusive. ADR is known to exert toxicity on the glomeruli and directly on renal tubules, leading to tubulointerstitial inflammation and fibrosis. Consequently, ADR administration results in elevated BUN and Cre levels, culminating in end-stage renal failure. In the ADR nephropathy model utilized in this study, direct tubular damage is also thought to occur due to ADR, suggesting that the FVB strain demonstrates a strong sensitivity to direct tubular damage caused by ADR.

A comparative analysis was performed after ADR administration to assess the severity of chronic nephropathy in the different strains at day 28. This study revealed that FVB-*Prkdc^R2140C^* mice had significantly increased urinary Alb/Cre and a higher percentage of fibrosis compared to their B6-*Prkdc^R2140C^* counterparts, indicating a profound susceptibility to CKD within the FVB-*Prkdc^R2140C^* strain. CKD, particularly in its early stages, can present with a pathology that reflects the primary disease; however, the specificity of the lesions often remains unclear. Nevertheless, fibrosis is a pervasive pathologic feature observed in many CKD cases [34]. By 28 days after ADR administration, the histopathological features observed in the FVB-*Prkdc^R2140C^* mice were similar to those observed in human chronic kidney disease, particularly interstitial fibrosis. This observation highlights that the FVB-*Prkdc^R2140C^* strain can be utilized as a robust model for elucidating the complexities and therapeutic targets of human CKD and confirms its validity and relevance in the study of renal pathology.

Subsequent RNA-seq analysis elucidated genes with altered expression across strains. Principal component analysis (PCA) and clustering analyses revealed that ADR nephropathy leads to significant differences in gene expression profiles between strains. Meanwhile, the severity of ADR nephropathy was shown to be influenced by modifier genes, with a notable strain difference observed between B6 and BALB, attributed to causative genes located between *D9Mit182* and *D9Mit229* on chromosome 9 of B6J, as revealed by previous research [14]. Consequently, examining the genes within this locus that showed significant expression changes between B6 and BALB identified eight genes: *Mmp7*, *Casp4*, *Mmp10*, *Casp12*, *Mmp27*, *Casp1*, *Mmp12*, and *Birc3*. qRT-PCR subsequently confirmed the reproducibility of these findings for these eight genes.

qRT-PCR analysis revealed a significant increase in the expression of *Mmp10*, *Mmp7*, *Mmp27*, *Casp4*, and *Birc3* in ADR-treated B6-*Prkdc^R2140C^* mice compared to ADR-treated BALB/c mice. Matrix metalloproteinases (MMPs) are proteolytic enzymes that contain a metal ion in their active site and are involved in the degradation of the extracellular matrix, including collagen, proteoglycans, and elastin, as well as the processing of bioactive substances and the degradation of cell surface expressed proteins [35]. Matrix metalloproteinase-10 (MMP-10), a zinc-dependent endopeptidase, can degrade various extracellular matrices and other protein substrates. The expression of MMP-10 is induced in both acute kidney injury (AKI) and CKD [36]. At different stages of renal injury, MMP-10 performs diverse functions by cleaving various bioactive substrates, such as heparin-binding EGF (HB-EGF), zonula occludens-1 (ZO-1), and pro-MMP-1, -7, -8, -9, -10, and -13. MMP-10 may contribute to protecting against AKI by enhancing EGFR signaling [37], thereby promoting tubular cell survival and proliferation after injury, suggesting a protective role against the progression of ADR nephropathy. Although Mmp27 is physiologically expressed in the kidney, its association with renal injury has not been reported. Casp4 and Birc3 are upregulated in tubular cells damaged by ischemia-reperfusion in an AKI model, as revealed by single-cell RNA sequencing analysis of proximal tubules. Baculoviral IAP repeat containing 3 (BIRC3), a member of the inhibitor of apoptosis protein family, attenuates apoptosis by inhibiting caspase activity [38]. On the other hand, caspase 4 (CASP4) is known to induce cellular apoptosis under conditions of endoplasmic reticulum stress [39]. In the B6 mouse strain, the presence of BIRC3 can neutralize CASP4-driven apoptosis, suggesting a functional protective mechanism against renal injury in these mice.

In ADR-treated B6-*Prkdc^R2140C^* mice, the expression of *Mmp12* was significantly reduced compared to ADR-treated BALB/c mice. There have been reports linking *Mmp12* to kidney damage associated with obesity. MMP-12, produced by macrophages infiltrating the glomeruli, contributes to the degradation of type IV collagen and fibronectin, and MMP-12 deficiency results in reduced glomerulosclerosis and fibrosis [40]. Given the significant increase in fibrotic area observed in B6-*Prkdc^R2140C^* mice compared to BALB/c, the reduced expression of this gene may contribute to the resistance of B6 kidneys to fibrosis.

In ADR-treated B6-*Prkdc^R2140C^* mice, *Mmp7*, which had significantly higher expression compared to both ADR-treated BALB/c and FVB-*Prkdc^R2140C^* mice, was reported to have a protective effect against AKI. Evidence from studies using MMP-7 knockout mice supports this notion, as these mice showed increased mortality rates, higher serum Cre levels, and more severe histologic damage following ischemic or nephrotoxic insults [41]. Thus, it can be inferred that upregulation of *Mmp7* in B6-*Prkdc^R2140C^* mice subjected to ADR may ameliorate tubular damage compared to the damage observed in their BALB/c and FVB-*Prkdc^R2140C^* counterparts. This study identifies *Mmp7*, *Mmp10*, and *Mmp12* as key genes contributing to the resistance of the B6 strain to ADR-induced tubular injury. The significant difference in the expression of these genes suggests a genetic basis for the observed phenotypic resistance. However, further investigation, including protein expression analysis, is essential to confirm these findings. Identifying and characterizing these resistance genes holds great promise for advancing our understanding of the molecular mechanisms underlying resistance to kidney disease. Such knowledge may pave the way for discovering novel therapeutic targets and developing innovative pharmacological strategies to mitigate renal injury.

## 5. Conclusions

In conclusion, this study established congenic mice by integrating the *Prkdc^R2140C^* from B6- *Prkdc^R2140C^* into the FVB/N genetic background, allowing a comparative analysis of the severity of ADR nephropathy in the FVB, B6, and BALB/c mice strains. Our results show that the FVB strain is more susceptible to ADR nephropathy than the B6 and BALB/c strains, highlighting a specific genetic predisposition to ADR-induced kidney damage. Our findings provide a basis for understanding how different strains respond to ADR nephropathy and suggest that *Mmp7*, *Mmp10*, and *Mmp12* may play a role in making the B6 strain more resistant to tubular cell damage. Although our study represents a significant step forward, it also highlights the need for further in-depth analyses to unravel the intricate genetic underpinnings contributing to the differential susceptibility and resistance observed among these mouse strains. Ultimately, unraveling these genetic factors may pave the way for novel therapeutic approaches to mitigate CKD, offering hope for improved treatment strategies in human kidney disease.

## Figures and Tables

**Table 1:** The primers used for qRT-PCR detection.

Gene Names	Forward Primer (5′-3′)	Reverse Primer (5′-3′)
*ActB*	cagaaggagattactgctctggct	tactcctgcttgctgatccacatc
*Birc3*	cagaggtcattgctggcgtt	tggtcggttttactgctaggc
*Casp1*	acaaggcacgggacctatg	tcccagtcagtcctggaaatg
*Casp4*	gaagacttaggctacgatgtggtg	tgtctgatgtctggtgttctgag
*Casp12*	gcccatgtggagacagattt	atagtgggcatctgggtcag
*Mmp7*	cttacctcggatcgtagtgga	ccccaactaaccctcttgaagt
*Mmp10*	tccaggaattgagccacaag	gggtcaaactcgaactgtgat
*Mmp12*	gcagtcctctatttcaaaagacac	ccaaaccagcttgtacttttcaatg
*Mmp27*	aggataataaagtgcttcccagga	aagaaatagaggaatccattatgttgg

## Data Availability

The detailed data of the current study are available from the corresponding authors upon reasonable request.

## Supplementary Materials

The following supporting information can be downloaded at: https://www.mdpi.com/article/10.3390/genes15040456/s1, Table S1: Differentially expressed genes.
